# Endoscopic nasobiliary drainage improves jaundice attack symptoms in benign recurrent intrahepatic cholestasis: A case report

**DOI:** 10.3892/etm.2012.814

**Published:** 2012-11-16

**Authors:** NORITAKA WAKUI, MITSURU FUJITA, NOBUYUKI OBA, YOSHIYA YAMAUCHI, YUKI TAKEDA, NOBUO UEKI, TAKAFUMI OTSUKA, SHUTA NISHINAKAGAWA, SAORI SHIONO, TATSUYA KOJIMA

**Affiliations:** 1 Departments of Internal Gastroenterology, Tokyo Rosai Hospital, Tokyo 143-0013, Japan; 2 Pathology, Tokyo Rosai Hospital, Tokyo 143-0013, Japan

**Keywords:** benign recurrent intrahepatic cholestasis, endoscopic nasobiliary drainage, arrival time parametric imaging, contrast-enhanced sonography, Sonazoid

## Abstract

A 66-year-old male with unbearable pruritus and jaundice was admitted for detailed examination. Blood tests on admission showed increased bilirubin with a dominant direct fraction. Ultrasonography and computed tomography performed subsequent to admission showed no narrowing or distension of the bile ducts. As the jaundice symptoms were not improved by the oral administration of ursodeoxycholic acid (300 mg/day) that had been started immediately after admission, endoscopic retrograde cholangiopancreatography (ERCP) was performed on hospital day 14. This also showed no abnormalities of the bile ducts. After considerating its potential effects for improving jaundice, endoscopic nasobiliary drainage (ENBD) was performed on the same day and was followed by immediate improvements in pruritus and jaundice. Detailed examinations were performed to identify the cause of the jaundice, which was suspected to be viral hepatitis, autoimmune hepatitis or drug-induced liver injury, however, there were no findings suggestive of any of these conditions. Following a further increase in bilirubin levels, confirmed by additional blood tests, a liver biopsy was performed. Histological findings were consistent with the histological features of benign recurrent intrahepatic cholestasis (BRIC). Although ursodeoxycholic acid is used as a first-line treatment in most cases of BRIC, ENBD should also be considered for patients not responding to this treatment.

## Introduction

In 1959, Summerskill and Walshe first reported benign recurrent intrahepatic cholestasis (BRIC) as a rare disease that did not progress to cirrhosis despite recurrent jaundice of an unknown origin (1). BRIC is an autosomal recessive disorder (2) caused by the mutation of two hepatic transporter genes: the *ATP8B1* gene, coding for familial intrahepatic cholestasis-1 (FIC1; BRIC type 1) and the *ABCB11* gene, coding for the bile salt export pump (BSEP; BRIC type 2) (3,4). Pruritus and jaundice are the only subjective symptoms and cholestasis generally improves within a few months. Although the level of serum alkaline phosphatase increases significantly, the level of γ-glutamyltranspeptidase remains within the normal range.

Unlike progressive familial intrahepatic cholestasis (PFIC), which is caused by the same genes and progresses to chronic intrahepatic cholestasis, BRIC has a fair prognosis without any progression to cirrhosis (2). In Japan, only 20 cases of BRIC type 1 have been reported thus far (5).

In the present study a difficult case of BRIC with prolonged jaundice was reported. Endoscopic nasal biliary drainage (ENBD) was performed to discharge the bile outside the body, which immediately improved the jaundice and pruritus.

## Case report

A 66-year-old male developed pruritus of an unprecedented intensity and also experienced general malaise and white stool in early May 2012. The patient presented to the Tokyo Rosai Hospital after his wife remarked on his yellow eyes. The patient’s alcohol consumption was 3 gō of Japanese sake (equivalent to 66 g ethanol) per day, 5 days per week until he stopped drinking in April 2012. There was no history of smoking. His prior medical history included cerebral infarction at age 48 and a cholecystectomy for cholecystitis at age 62. There was no significant family history reported. The patient was taking the following oral medication: pitavastatin 1 mg/day, olmesartan 20 mg/day, aspirin 81 mg/day, cilnidipine 15 mg/day, nicergoline 15 mg/day and famotidine 40 mg/day. At presentation, he had a clear sensorium, with a blood pressure of 138/90 mmHg, a pulse rate of 60 beats/min (non-arrhythmic) and a body temperature of 36.0°C. The palpebral conjunctiva was not anemic while the bulbar conjunctiva was yellow. Heart and breath sounds were clear. The abdomen was flat and soft with no tenderness or rebound tenderness. The liver and spleen were impalpable. Laboratory findings at presentation included increased bilirubin levels with a direct dominant fraction, a T-Bil of 12.6 mg/dl and a D-Bil of 9.7 mg/dl, an increased total bile acid level of 101.5 μmol/l and mild hepatic impairment as indicated by an AST of 29 IU/l and an ALT of 49 IU/l (Table I).

**Figure 1.**
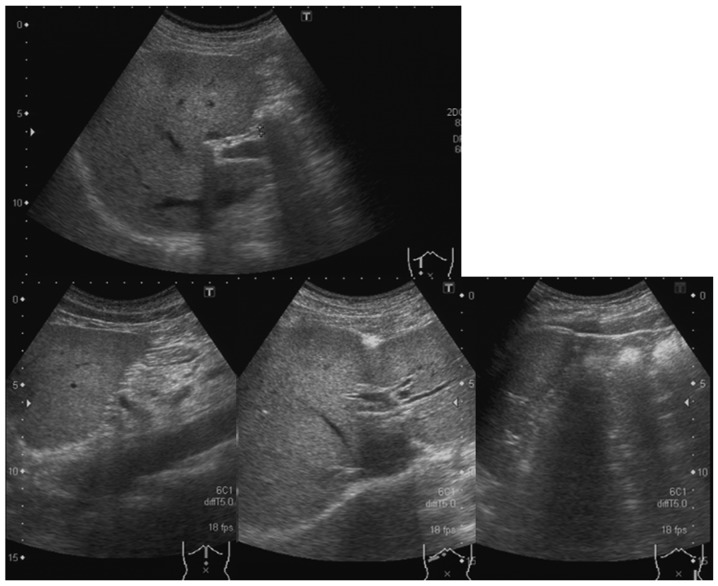
B-mode abdominal ultrasound (US) image on hospital day 2 showing fatty liver-like parenchyma with (A) patchy bright areas. (A,B) No hepatomegaly, (D) splenomegaly, (A–C) mass lesion, (A,C) ascites or distention of the intrahepatic or common bile ducts were observed.

**Figure 2.**
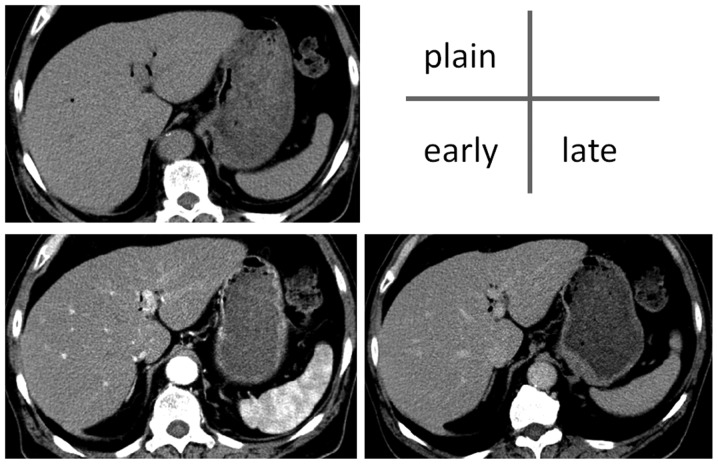
Abdominal computed tomography (CT) images on hospital day 7 showing no hepatomegaly, splenomegaly, ascites or distention of the intrahepatic or common bile ducts.

The patient was subsequently admitted for a detailed examination into the causes of the jaundice. Abdominal ultrasonography (US) performed on hospital day 2 showed a fatty liver-like parenchyma with patchy bright areas. Neither hepatomegaly nor splenomegaly was observed and no mass lesions were observed in the liver. No ascites or distension of the intrahepatic or common bile ducts were apparent (Fig. 1). Abdominal computed tomography (CT) performed on hospital day 7 also revealed no hepatomegaly, splenomegaly, distension of the intrahepatic or common bile ducts or ascites (Fig. 2). Following a further increase in bilirubin levels confirmed by blood testing, Sonozoid-enhanced US with arrival time parametric imaging (At-PI), an imaging technique that has been shown to be effective in evaluating the degree of progression of hepatic lesions (3), was performed on hospital day 9.

### Contrast-enhanced US and At-PI

**Figure 3.**
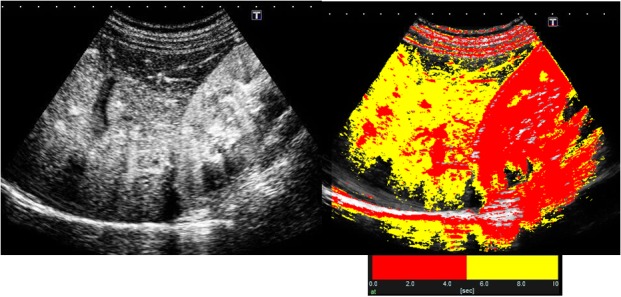
Contrast enhancement pattern of the liver parenchyma 10 sec after the arrival of the contrast medium, following a bolus infusion of Sonazoid via the median cubital vein. Sonazoid-enhanced images of the liver parenchyma with (right) and without (left) arrival-time parametric imaging (At-PI).

**Figure 4.**
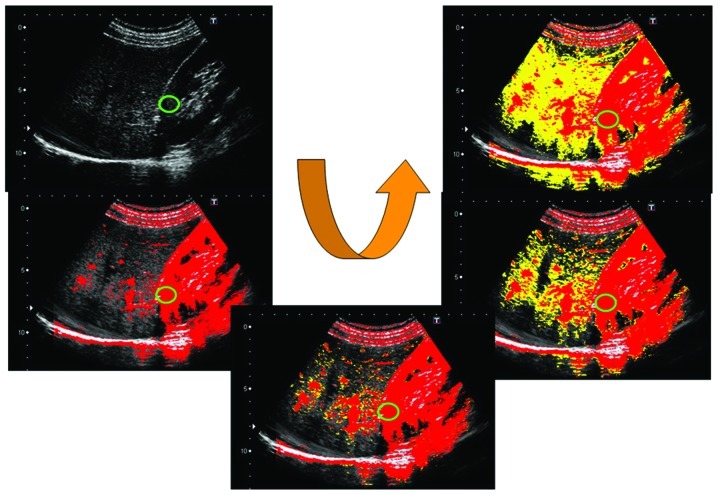
Procedure for At-PI. Subsequent to the region of interest (green circle) being set in the kidney parenchyma, the stored movie is played and arrival times are sequentially calculated for each liver parenchymal pixel, with a time point at which 80% of the ROI is enhanced by contrast medium defined as time 0. Then, a color map is automatically superposed on a B-mode image. Pixels with an arrival time of 0–5 sec are displayed in red and those with an arrival time of 5–10 sec in yellow.

The Toshiba SSA-790A (AplioXG; Toshiba Medical Systems, Otawara, Japan) US system was used with a 3.75-MHz convex probe (PVT-375BT). Imaging was performed with a mechanical index of 0.21. Images showing the liver parenchyma from the right inter-costal space to the S6 region of the right hepatic lobe and the right kidney all on a single screen were used for analysis. The focus was set at a depth of 8 cm to visualize the kidney. After imaging conditions were set, Sonazoid (perfluorobutane; GE Healthcare, Oslo, Norway) was infused at the recommended dose of 0.015 ml/kg via the median cubital vein. Images captured immediately after the 40-sec Sonazoid infusion were saved as raw data on the system’s hard disk drive. The At-PI calculation and drawing were performed with the saved movie data, using software provided with the system, as described previously (6). Briefly, subsequent to the region of interest (ROI) being set in the kidney parenchyma, the movie was played and arrival times were sequentially calculated for each liver parenchymal pixel, with the time point at which 80% of the ROI was enhanced by contrast medium defined as time 0. A color map was then automatically superposed on a B-mode image. The display colors were set according to user preference, and in the present study, color mapping was configured to display the arrival times of 0–5 sec in red and those of 5–10 sec in yellow (Figs. 3 and 4).

### Calculation of the ratio of red (ROR) area to the entire contrast-enhanced area

**Figure 5.**
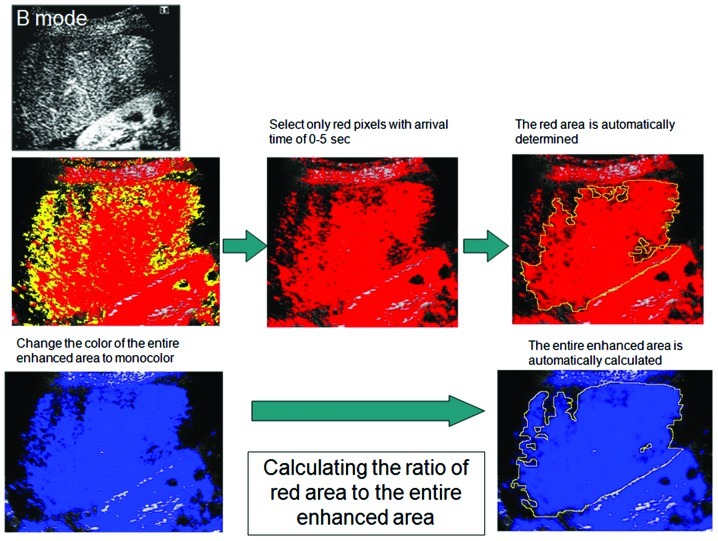
Procedure to calculate the ratio of red (ROR) area to the entire contrast-enhanced area of the liver parenchyma. From the obtained arrival-time parametric images (At-PI), the areas of the red-colored section of the liver parenchyma and the entire contrast-enhanced area were calculated using ImageJ.

**Figure 6.**
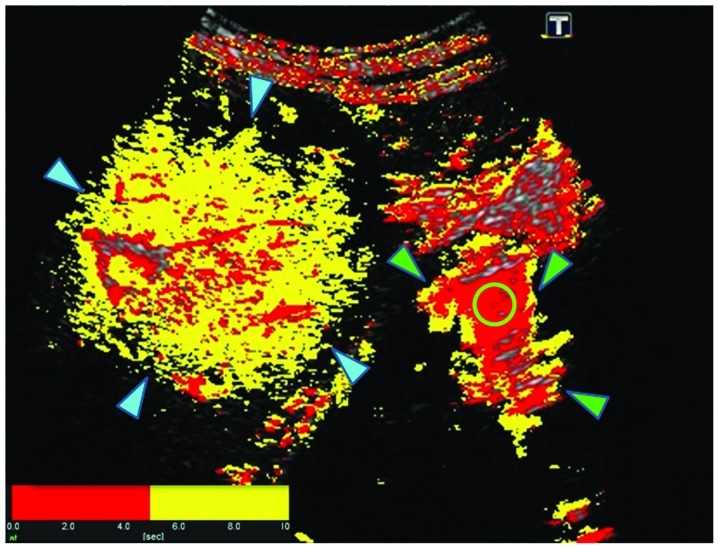
Sonazoid-enhanced ultrasonography (US) image with arrival time parametric imaging (At-PI) taken during an episode of jaundice (hospital day 9; Arrow heads: Blue indicate the liver and green indicate the kidney). The majority of the liver parenchyma was yellow, with a ratio of red (ROR) of 15.7%.

For a quantitative evaluation of the At-PI data obtained, the ratio of the area of red pixels with shorter arrival times to the entire contrast-enhanced area was calculated as the ROR using image analysis software ImageJ (version 1.42. Wayne Rasband, National Institutes of Health, Bethesda, MD, USA; Fig. 5). The ROR during an episode of jaundice (hospital day 9; T-Bil 19.3 mg/dl, D-Bil 15.2 mg/dl) was calculated as 15.7% (Fig. 6).

### Clinical course following admission

**Figure 7.**
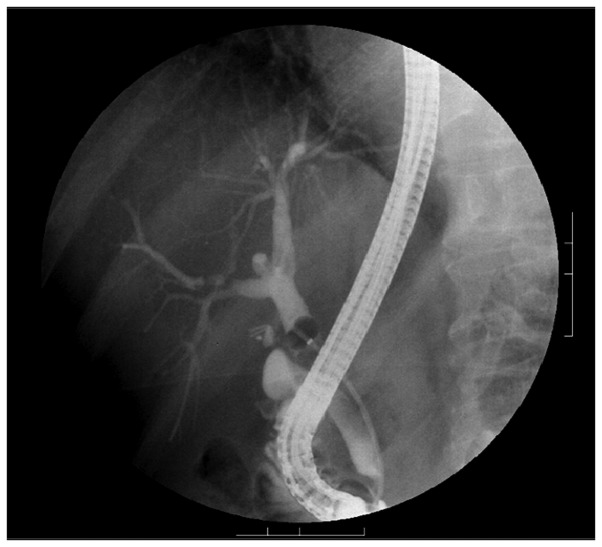
Endoscopic retrograde cholangiopancreatography (ERCP) image (hospital day 14) showing no apparent abnormality in the intrahepatic or common bile ducts.

**Figure 8.**
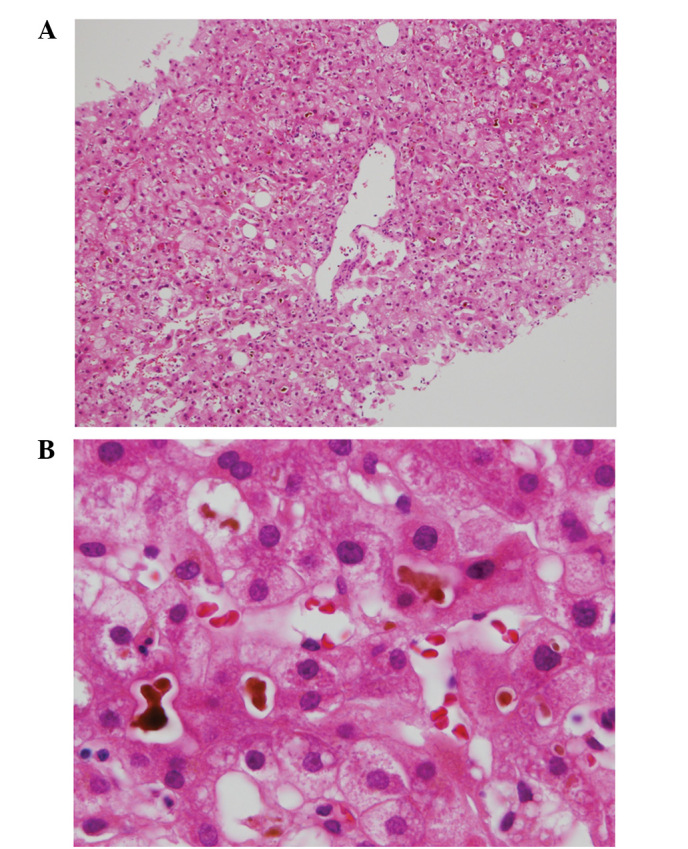
Histological images from the liver biopsy. (A) Bile deposition in the centrilobular hepatocytes and bile thrombus formation, with minimal inflammatory and fibrotic findings (H&E; ×40). (B) Multiple bile thrombi (H&E; ×400).

As the jaundice had not been improved by the oral administration of ursodeoxycholic acid (300 mg/day) that had been started immediately after hospital admission, endoscopic retrograde cholangiopancreatography (ERCP) was performed on hospital day 14 for direct scrutiny of the bile ducts. This again revealed no abnormalities*,* including distension or narrowing of the common or intrahepatic bile ducts (Fig. 7). After considering its potential effects for improving jaundice, ENBD was performed on the same day and was followed by an immediate improvement in jaundice and skin pruritus. Subsequently, a liver biopsy was performed on hospital day 27 for further examination of the liver. The biopsy revealed bile deposition in the centrilobular hepatocytes and bile thrombus formation, with minimal inflammatory and fibrotic findings (Fig. 8).

**Figure 9.**
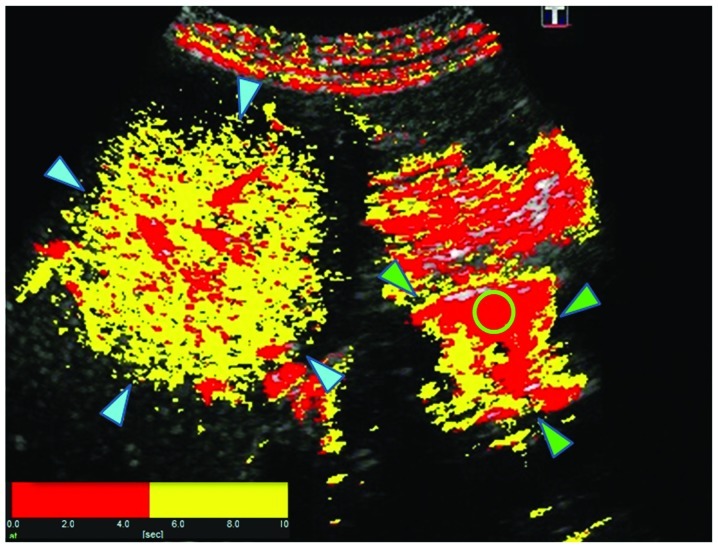
Sonazoid-enhanced ultrasonography (US) image with arrival time parametric imaging (At-PI) when the patient was recovering from jaundice (hospital day 40; Arrow heads: Blue indicate the liver and green indicate the kidney). The majority of the liver parenchyma was yellow, with a ratio of red (ROR) of 11.6%.

**Figure 10.**
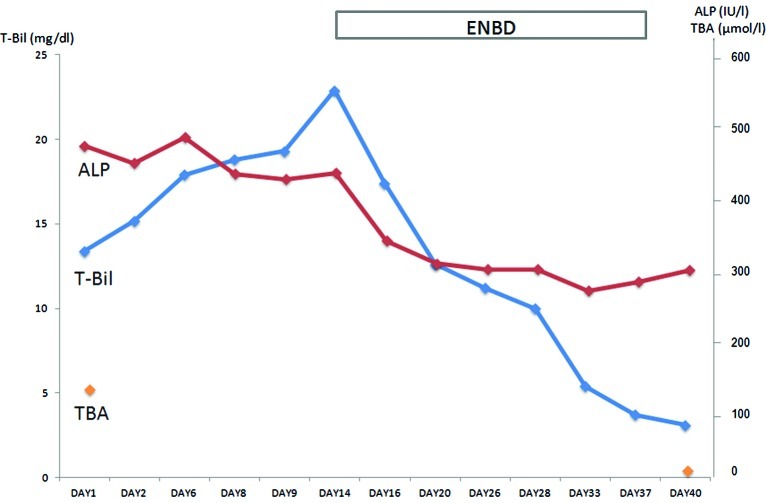
Clinical course subsequent to hospital admission. Jaundice was improved rapidly following endoscopic nasobiliary drainage (ENBD). TBA, total bile acids; T-Bil, total bilirubin; ALP, alkaline phosphatase.

Sonazoid-enhanced US with At-PI was performed as the patient was recovering from jaundice (hospital day 40: T-Bil, 3.1 mg/dl, D-Bil, 2.5 mg/dl). The ROR was calculated as 11.6%, which was not significantly different from the value obtained when the patient was jaundiced (Fig. 9). The patient was discharged well on hospital day 41. The patient’s clinical course following admission is shown in Fig. 10. Written informed patient consent was obtained from the patient. This study was performed with approval of the Ethics Committee at Tokyo Rosai Hospital (Tokyo, Japan).

## Discussion

BRIC is a rare condition that is clinically diagnosed according to five criteria: multiple episodes of jaundice with severe pruritus and laboratory findings suggestive of cholestasis; absence of factors known to be associated with cholestasis, including drugs and pregnancy; normal intrahepatic and extrahepatic bile ducts confirmed by direct cholangiography; liver biopsy demonstrating bile thrombus formation; and a symptom-free interval lasting several months to years (7). BRIC-1 is an autosomal recessive disorder caused by a mutation in the *ATP8B1* gene on chromosome 18q21 that encodes the FIC1 protein, a P-type ATPase. The gene is also known to be responsible for the development of PFIC. The FIC1 protein is localized to the cell membranes of the hepatocytes that form the bile canaliculi (8). Although the functions of the FIC1 protein have not been fully elucidated, one of its suggested functions is to flip phospholipids present in the outer layer of the lipid bilayer of cell membrane, such as phosphatidylserine, into the inner layer. Lack of the FIC1 protein leads to disruption of the lipid arrangement in the cell membrane exposed to the bile canaliculus and increases susceptibility to damage caused by hydrophobic bile acid, resulting in cholestasis (9). Recently, a new form of BRIC was reported with a mutation not in the *ATP8B1* gene but in the *ABCB11* gene on chromosome 2q24 (4). This has become known as BRIC-2. The *ABCB11* gene encodes the BSEP protein whose mutation is also known to cause PFIC-2. BSEP protein is localized to the cell membrane of hepatocytes exposed to the bile canaliculi and functions to transport bile acids from the hepatocytes into the canaliculi. Thus, it is difficult to clinically distinguish between the two types of BRIC associated with mutations in different genes. Moreover, although BRIC shares the same causative gene with PFIC, PFIC is characterized by persistent cholestasis that may progress to cirrhosis, whereas BRIC is characterized by recurrent but transient cholestasis that does not cause permanent damage to the liver.

BRIC is also characterized by non-age-related occurrences, recurrent episodes of pruritus and increased levels of bilirubin with a dominant direct fraction and blood TBA. Episodes may last from several weeks to months and usually resolve spontaneously. The severity of jaundice varies from mild to severe and the interval between the jaundice episodes varies from months to a decade or more (10,11). In the present study, the patient was under considerable stress from pressure at work and began suffering from malaise around April 2012. He then experienced pruritus of an unprecedented intensity and had white stool in early May 2012. The patient was informed by his wife that his eyes had become yellow. In the majority of BRIC cases, the initial clinical manifestation is of severe pruritus followed by an awareness of jaundice and white stool a few days later (12), which is similar to the present case. Another characteristic finding is a mild increase in ALT/AST (<3 times the normal level). In the present study, only mild increases were observed in these parameters, with an ALT of 49 IU/l and an AST of 29 IU/l. The absence of bile duct distension is another characteristic finding of BRIC. US, CT and ERCP were performed in the present study due to increased bilirubin levels with a dominant direct fraction on the blood tests at admission, but there were no abnormal findings that indicated bile duct narrowing. Detailed examinations to identify the cause of the jaundice were performed although viral hepatitis and autoimmune hepatitis were suspected. The patient was identified as negative for anti-HCV, anti-HBs, HBsAb, anti-nuclear antibodies (ANA) and anti-mitochondiral antibodies (AMA), indicating that hepatitis C/B and autoimmune hepatitis were unlikely. Drug-induced hepatitis was also suspected, but again ruled out as there had been no changes in the patient’s oral medication in the past year. Due to the persistent and progressive increase in bilirubin even after admission, a liver biopsy was also performed. This procedure revealed bile deposition in the centrilobular hepatocytes and bile thrombus formation, with minimal inflammatory and fibrotic findings. These findings were consistent with the characteristic features of BRIC (13).

The patient had not experienced symptoms of jaundice and pruritus prior to this occurrence and, although genetic analysis was not performed, the diagnosis of BRIC was made based on the clinical course and histological findings. Sonazoid-enhanced US with At-PI was also performed. This procedure allows for the degree of progression of a hepatic lesion to be evaluated by analyzing blood flow balance between the hepatic artery and portal vein, the two vessels supplying the liver. In the present study, the ROR was calculated as 15.7% during an episode of jaundice which was comparable to that of the normal liver (13.7%) as reported previously (6). This also suggested the absence of a progressive lesion in the liver and supported the diagnosis of BRIC, rather than PFIC. Moreover, the fact that there was no significant difference between the ROR calculated during the episode of jaundice and that calculated while the patient was recovering from jaundice also suggests that cholestasis in BRIC does not affect the portal vein/hepatic artery blood flow balance.

BRIC is associated with recurrent cholestasis but does not cause permanent damage to the liver, it therefore differs from PFIC which is associated with persistent cholestasis and progression to cirrhosis. This means that monitoring for the presence or absence of progression to cirrhosis is essential for the differential diagnosis between the two conditions. Sonazoid-enhanced US with At-PI may be performed non-invasively and repeatedly, unlike liver biopsies, and thus is useful in monitoring and identifying patients with early BRIC or PFIC.

Ursodeoxycholic acid is used as the first-line treatment for jaundice and pruritus in most cases of BRIC, but it is not necessarily effective (14). The efficacy of rifampicin against cholestasis (15) and the usefulness of cholestyramine have also been documented, but certain studies suggest that these agents have a transient effect (16). While a study has suggested that medical therapy for BRIC has insufficient effects, the usefulness of ENBD has been demonstrated by Stapelbroek *et al*(17), who performed ENBD for bile drainage in 3 patients with BRIC and reported that blood TBA levels (100–200 μmol/l pretreatment) decreased to almost normal levels 3 days after the insertion of an ENBD tube. A resolution of pruritus was also reported in all patients. The present study also showed significant decreases in the patient’s TBA and total bilirubin levels in ∼1 week following ENBD tube insertion and additionally that these levels were maintained almost within the normal ranges even subsequent to tube removal. ENBD may therefore be considered as highly effective in the treatment of jaundice and pruritus in BRIC. The effectiveness of ENBD against cholestasis in BRIC may be explained by evidence showing that it forces bile drainage, blocks the enterohepatic circulation and that subsequent reduction in the bile acid pool results in restoring the function of bile excretion transporters. In obstructive jaundice, an internal fistula is usually created by bile duct stenting. In cases of BRIC, however, bile duct stenting is ineffective as there is no narrowing or other abnormality in the intra- or extrahepatic bile ducts and bile drainage cannot be achieved by an internal fistula. It remains unclear as to how transporter function is restored by ENBD. Stapelbroek *et al*(17) noted increased levels of phospholipids other than phosphatidylcholine, particularly sphingomyelin, in the bile drained by ENBD and suggested that a disrupted phospholipid gradient may have been restored as a result of the bile acid pool being reduced.

In conclusion, although a rare condition, the possibility of BRIC should be considered and an appropriate workup should be performed when patients present with severe pruritus, an increase in direct bilirubin and a mild increase in transaminase levels. The use of ENBD should be considered for the treatment of BRIC if jaundice is not improved by the first-line treatment of ursodeoxycholic acid.

In suspected cases of early BRIC or PFIC, Sonazoid-enhanced US with At-PI may be useful in distinguishing between the two conditions as it is minimally invasive and may be performed repeatedly.

## Figures and Tables

**Table I:** Laboratory test results on admission

Diagnostic blood tests	Results
Biochemistry	
CRP	0.4 mg/dl
Na	138 mEq/l
K	3.7 mEq/l
Cl	105 mEq/l
TP	7.1 g/dl
Alb	4.4 g/dl
T-Bil	12.6 mg/dl
D-Bil	9.7 mg/dl
AST	29 IU/l
ALT	49 IU/l
LDH	174 IU/l
ALP	446 IU/l
GGT	154 IU/l
T-Cho	197 mg/dl
TG	466 mg/dl
BUN	12 mg/dl
Cr	0.56 mg/dl
BS	136 mg/dl
HbA1c	6.0%
PT%	119%
PT-INR	0.93
Hematology	
WBC	8100/μl
RBC	453×10^4^/μl
Hgb	14.6 mg/dl
Hct	41.9%
PLT	26.3×10^4^/μl
Serology	
anti-HCV	(−)
HBsAg	(−)
anti-HBs	(−)
ANA	(−)
AMA	(−)
p-ANCA	(−)
IgG	981 mg/dl
IgA	263 mg/dl
IgM	97 mg/dl
TBA	101.5 μmol/l

CRP, C-reactive protein; TP, total protein; Alb, albumin; T-Bil, total bilirubin; D-Bil, direct bilirubin; AST, aspartate aminotransferase; ALT, alanine aminotransferase; LDH, lactate dehydrogenase; ALP, alkaline phosphatase; GGT, gamma-glutamyltranspeptidase; T-Cho, total cholesterol, TG, triglyceride; BUN, blood urea nitrogen; Cr, creatinine; BS, blood sugar; HbA1c, hemoglobin A1c; PT%, prothrombin time %; PT-INR, prothrombin time International normalized ratio; WBC, white blood cell; RBC, red blood cell; Hgb, hemoglobin; Hct, hematocrit; PLT, platelet; HCV, hepatitis C virus; HBsAg, hepatitis B surface antigen; ANA, anti-nuclear antibody; AMA, anti-mitochondrial antibody; P ANCA, perinuclear anti-neutrophil cytoplasm antibodies; IgG, immunoglobulin G; IgA, immunoglobulin A; IgM, immunoglobulin M; TBA, total bile acids.

## Abbreviations:

At-PI: arrival time parametric imaging
US: ultrasonography
CT: computed tomography
AMA: anti-mitochondrial antibody
ANA: anti-nuclear antibody
p-ANCA: perinuclear anti-neutrophil cytoplasm autoantibodies
TBA: total bile acids
BRIC: benign recurrent intrahepatic cholestasis
ENBD: endoscopic nasobiliary drainage
ROR: ratio of red
ROI: region of interest
PFIC: progressive familial intrahepatic cholestasis
BSEP: bile salt export pump
